# Preoperative sleep disturbance as a risk factor for moderate-to-severe postoperative pain in hemifacial spasm patients: a prospective cohort study

**DOI:** 10.3389/fneur.2025.1670952

**Published:** 2025-10-22

**Authors:** Qun Gao, Shanshan Ma, Bo Hei, Bin Wang, Manyu Sun, Shuju Jia, Jingru Zhou

**Affiliations:** ^1^Department of Neurosurgery, Peking University People’s Hospital, Beijing, China; ^2^Department of Public Health, Affiliated Hospital of Jining Medical University, Jining, China

**Keywords:** hemifacial spasm, preoperative sleep quality, microvascular decompression, postoperative pain, clinical psychology

## Abstract

**Objective:**

This study aims to examine the impact of preoperative sleep disturbances on postoperative pain levels among individuals undergoing hemifacial spasm (HFS) surgery. The findings will establish a theoretical foundation for developing targeted interventions to ameliorate postoperative pain management in this patient population.

**Methods:**

Hemifacial spasm patients who were treated and operated on at Peking University People’s Hospital from October 2023 to February 2024 were selected as participants via convenience sampling. Sleep quality was quantitatively assessed using the Pittsburgh Sleep Quality Index (PSQI), while postoperative pain severity was measured with the Numerical Rating Scale (NRS). Perioperative clinical data were systematically recorded at postoperative day 1, 3, and 7 (POD 1, 3, and 7). Univariate and multivariate regression analyses were subsequently conducted to identify predictors of moderate-to-severe pain persisting at the 1-week postoperative timepoint.

**Results:**

A cohort of 120 patients diagnosed with hemifacial spasm participated in this study. Based on preoperative Pittsburgh Sleep Quality Index (PSQI) scores, participants were stratified into two groups: a low-sleep-quality group (PSQI ≥ 7, *n* = 25) and a high-sleep-quality group (PSQI < 7, *n* = 95). At POD 7, a total of 22 patients (18.33%) developed moderate-to-severe pain (NRS ≥ 4). Univariate analysis revealed that the pain incidence was significantly higher in the poor sleep quality group (PSQI ≥ 7) compared to the good sleep quality group (44.0% vs. 11.58%, *p* = 0.006). After adjusting for confounding variables, multivariate analysis confirmed that preoperative sleep disturbance (PSQI score) was an independent risk factor for postoperative pain (adjusted OR = 1.368, 95% CI: 1.154–1.621, *p* < 0.001). Each 1-point increase in PSQI score was associated with a 36.8% increase in pain risk. Sensitivity analysis using dichotomized PSQI variables (cut-off values ≥5 or ≥7) yielded consistent results, showing significantly increased pain risk in the poor sleep quality group (OR = 6.263 and 6.419, respectively, both *p* = 0.001), supporting the robustness of the primary analysis findings.

**Conclusion:**

Preoperative sleep disturbances elevate the risk of patients with hemifacial spasm experiencing moderate-to-severe pain postoperatively. Therefore, proactive management of sleep disturbances prior to surgery represents a valuable strategy for enhancing postoperative analgesia in this patient population. However, the generalizability of these findings is limited to HFS patients undergoing MVD, and further validation in broader surgical cohorts is warranted.

## Introduction

Hemifacial spasm (HFS) is a neurological disorder characterized by involuntary paroxysmal contractions of unilateral facial muscles, severely impacting patients’ quality of life and mental health (1). The condition is often associated with neurovascular compression of the facial nerve, leading to not only motor symptoms but also persistent neuropathic discomfort and pain in a subset of patients. Currently, microvascular decompression (MVD) is widely recognized as a definitive treatment with well-established efficacy (2). However, postoperative pain remains a common complication, potentially prolonging hospitalization and reducing patient satisfaction. In recent years, perioperative management research has gradually shifted from solely focusing on surgical techniques to multidimensional optimization of patients’ overall condition. Among these efforts, preoperative sleep disturbances, as a modifiable risk factor, have attracted increasing attention for their role in chronic pain and postoperative recovery (3, 4). Given the overlapping pathways involving sleep modulation, central sensitization, and neurogenic pain mechanisms, understanding the interplay between sleep quality and pain outcomes in HFS patients—a population inherently susceptible to neural dysfunction—holds particular relevance for the field of headache and neurogenic pain. Nevertheless, the correlation between preoperative sleep quality and postoperative pain in HFS patients has not been systematically explored, leaving a critical research gap that hinders the precision development of perioperative intervention strategies.

Sleep disturbances are prevalent among patients with hemifacial spasm (HFS), likely attributed to factors such as frequent facial muscle spasms disrupting sleep rhythms and psychological stress induced by the chronic condition (5). Chronic sleep impairment not only diminishes patients’ quality of life but may also compromise immune function and neurological recovery (6). Existing studies suggest that sleep disturbances exacerbate pain perception through mechanisms such as neuroinflammation activation and hypothalamic–pituitary–adrenal (HPA) axis dysregulation, while chronic pain conversely disrupts sleep patterns, forming a self-perpetuating cycle (7). In neurosurgical practice, the impact of preoperative sleep quality on postoperative pain has been partially validated in spinal surgery and brain tumor patients (8). However, research targeting HFS—a distinct patient population—remains lacking. Furthermore, HFS patients, burdened by long-term disease-related distress, often exhibit comorbid anxiety and depression, which may intensify the interplay between sleep disturbances and pain (9). Clarifying the association between preoperative sleep disturbances and postoperative pain in HFS patients will not only elucidate underlying biological mechanisms but also provide a theoretical foundation for developing personalized analgesic and sleep management protocols in clinical settings.

This prospective cohort study enrolled patients with HFS scheduled for MVD surgery to explore the correlation between preoperative sleep disturbances and postoperative pain. The Pittsburgh Sleep Quality Index (PSQI) and the Numerical Rating Scale (NRS) were used to assess sleep quality and pain, respectively. A multiple regression model was employed to analyze the independent association.

Consequently, this prospective cohort study was conducted to examine the association between preoperative sleep disturbances and postoperative pain levels in individuals undergoing microvascular decompression for hemifacial spasm. The findings aim to inform perioperative care strategies, ultimately optimizing pain management protocols for affected patients with coexisting sleep disruption.

## Materials and methods

### Study subjects

A total of 120 patients who had undergone MVD due to hemifacial spasm in Peking University People’s Hospital from October 2023 to February 2024 were selected as the research subjects. All patients gave informed consent and were approved by the hospital ethics committee (no. 2019PHB225-01). All surgeries were performed by the same surgical team.

### Inclusion criteria

(1) Subjects aged ≥18 years.
(2) Diagnosis of hemifacial spasm confirmed by clinical presentation (symptoms and signs) and neuroimaging (10).
(3) Absence of significant communication impairment.

### Exclusion criteria

(1) History of psychiatric illness, cognitive impairment, language disorders, or other conditions adversely affecting sleep assessment.
(2) Unwillingness or inability to provide informed consent or participate in study procedures.
(3) Insufficient preoperative clinical documentation.
(4) Missing key postoperative follow-up data.

### Research tool

The Pittsburgh Sleep Quality Index (PSQI) is a validated instrument for assessing sleep disturbances, psychological comorbidities, and overall sleep quality in general populations, demonstrating strong psychometric properties (11, 12). The PSQI is composed of 18 items that assess seven sleep dimensions (including sleep quality, time to fall asleep, duration, efficiency, disturbances, medication use, and daytime dysfunction). Each dimension scores 0–3 points, contributing to a global score ranging from 0 to 21. The primary analysis utilized the global PSQI score, as the study aimed to investigate the overall impact of subjective sleep quality on postoperative pain rather than the contribution of specific sleep components. The PSQI was administered to all participants within 3 days prior to surgery. This assessment captured the subjective sleep quality of patients over the preceding 1-month period, as is standard for this instrument. Its applicability extends to Chinese patient cohorts (11, 13–16). Subjects with a cumulative PSQI score of <7 were defined as having good sleep quality, and those with a score ≥7 as having poor sleep quality.

### Grouping

Preoperative sleep status in hemifacial spasm patients was assessed using the Pittsburgh Sleep Quality Index (PSQI). Participants were stratified into two groups based on established cutoff scores: a poor sleep quality group (PSQI ≥ 7, *n* = 25) and a good sleep quality group (PSQI < 7, *n* = 95). Demographic characteristics and longitudinal postoperative outcomes—including pain levels and sleep parameters—were systematically recorded. This methodology facilitated investigation of preoperative sleep quality’s influence on postoperative analgesic outcomes following microvascular decompression repair.

### Numeric rating scale

Pain intensity was quantified by two independent assessors using the Numerical Rating Scale (NRS). This instrument defines “0” as absence of pain and “10” as the most excruciating, incapacitating pain imaginable. Scores were stratified into clinically meaningful categories: mild (1–3), representing tolerable discomfort; moderate (4–6), indicating distressing pain interfering with sleep; and severe (7–10), signifying intense, unbearable suffering requiring intervention (17). Patients can choose any number from “0 to 10” to express their pain severity by fully feeling their pain. The evaluation time points were postoperative day 1 (POD 1), day 3 (POD 3), and day 7 (POD 7). The pain score at 1 week was defined as the NRS score on POD7. The nursing staff who assessed postoperative pain using the NRS were blinded to the patients’ preoperative PSQI scores and their corresponding sleep quality group assignment. Body temperature was routinely monitored at least twice daily during the postoperative period.

### Anesthesia and perioperative management

All patients received standardized general anesthesia and perioperative care. Both patient groups received the same anesthesia protocol and perioperative care. Anesthetic induction and endotracheal intubation were achieved using intravenous etomidate (0.2–0.3 mg/kg), oxycodone (0.1–0.15 mg/kg), and rocuronium (0.6–1.0 mg/kg). During the operation, anesthesia was maintained with remifentanil (0.05–0.2 μg/kg/min) and propofol (4–6 mg/kg/h) to keep the bispectral index between 45 and 55. Additional rocuronium was given intermittently to ensure muscle immobility. Standard monitoring throughout surgery encompassed electrocardiogram (ECG), invasive arterial pressure, pulse oximetry (SpO₂), and end-tidal carbon dioxide (PetCO₂). Hemodynamic stability was maintained intraoperatively, with blood pressure and heart rate fluctuations generally controlled within 20% of baseline values. PetCO₂ was regulated at 35–45 mmHg. A triple-drug prophylactic antiemetic regimen, comprising 1 mg droperidol, 40 mg methylprednisolone, and 5 mg tropisetron hydrochloride, was administered intravenously to all patients intraoperatively. Following the return of adequate spontaneous breathing, intravenous 1 mg neostigmine, 0.5 mg atropine, and 0.5 mg flumazenil were given for reversal. After the procedure, patients were admitted to the PACU for continued observation and postoperative care. They were later moved to the ward upon achieving a Steward score greater than 4.

### Data collection

The major endpoint of this study was the proportion with NRS ≥ 4 at POD7 (18–20). Trained nursing staff administered all study instruments following a standardized protocol. Prior to participation, comprehensive pre-administration briefings explained the research purpose, instrument significance, and completion procedures. Written informed consent was obtained from both participants and legal proxies before questionnaire distribution. Assessments were primarily self-administered by research subjects. During data collection, investigators provided clarification adhering to standardized scripts when participants required assistance. For individuals unable to complete forms independently, designated proxies or researchers documented responses via structured interview.

### Statistical analysis

Data analysis was performed using SPSS Statistics version 25.0. Continuous variables are presented as mean ± standard deviation (*x̄* ± S). Group comparisons employed independent *t*-tests or Mann–Whitney U tests based on distributional assumptions. Categorical data are expressed as frequencies (percentages) and analyzed using chi-square tests. To identify factors associated with moderate-to-severe pain (NRS ≥ 4) on postoperative day 7, univariate analysis was first conducted to screen potential predictive variables (inclusion criterion: *p* < 0.15). Subsequently, the selected variables along with clinically relevant *a priori* variables (including age, gender, BMI, comorbidities, smoking history, and surgical details) were incorporated into a multivariate binary logistic regression model to control for confounding effects and identify independent risk factors. Results are expressed as odds ratios (OR) with 95% confidence intervals (CI), and a *p*-value < 0.05 was considered statistically significant.

## Results

### Comparison of general data of the two groups of patients

As shown in Table 1, baseline characteristics—including age, gender, body mass index (BMI), education level, marital status, smoking history, and alcohol consumption, etc., exhibited no statistically significant differences (*p* > 0.05) between the high and poor sleep quality groups.

**Table 1:** Comparison of general data of patients in two groups (*n* = 120).

Characteristics	Poor sleep quality group (*n* = 25)	Good sleep quality group (*n* = 95)	*p*
Age	53.44 ± 9.64	53.39 ± 11.68	0.984
Gender (M)	7 (28.0%)	28 (29.5%)	0.99
BMI	24.24 ± 3.11	25.36 ± 3.34	0.134
Educational level			>0.05
Primary education	11	28	
Junior high education	9	43	
Senior high school	3	18	
College degree or above	2	6	
Marital status	24 (96.0%)	94 (98.9%)	0.375
Smoking	5 (20%)	12 (12.6%)	0.346
Drinking	3 (12%)	8 (8.4%)	0.696
Comorbidity	10 (40.0%)	43 (45.3%)	0.659
Side (left)	11 (44%)	48 (50.5%)	0.655
Responsible artery			>0.05
AICA	15	71	
PICA	7	16	
VA	2	5	
BA	1	3	

AICA, anterior inferior cerebellar artery; PICA, posterior inferior cerebellar artery; VA, vertebral artery; BA, basilar artery.

### Pain scores of patients in two groups at different time points

Postoperative pain scores differed significantly between the high and poor sleep quality groups at all measured intervals (*p* < 0.05). As presented in Table 2, patients reporting better sleep quality demonstrated consistently lower pain levels at POD 1, POD 3, and POD 7 compared to those with poorer sleep quality.

**Table 2:** Pain scores of patients in two groups at different time points (*n* = 120).

Variable	Poor sleep quality group (*n* = 25)	Good sleep quality group (*n* = 95)	*p*
Postoperative day 1	5.32 ± 1.49	3.35 ± 1.09	0.000
Postoperative day 3	4.32 ± 1.38	3.06 ± 1.23	0.000
Postoperative day 7	3.44 ± 1.53	1.92 ± 1.27	0.000

### The pain degree and analgesic drugs use in two groups at postoperative day 3

As detailed in Table 3, statistically significant intergroup differences were observed in pain severity and analgesic requirements (*p* < 0.05). Specifically, the group with better sleep quality exhibited fewer cases of moderate-to-severe pain at postoperative day 3 compared to the poorer sleep quality group. Furthermore, the proportion of patients requiring analgesic medication (oral loxoprofen sodium, once daily for three consecutive days) was significantly higher in the poor sleep quality group than in the good sleep quality group.

**Table 3:** Pain degree of patients in two groups at postoperative day 3 (*n* = 120).

Items	Poor sleep quality group (*n* = 25)	Good sleep quality group (*n* = 95)	*p*
Moderate-to-severe pain	14 (56.0%)	12 (12.6%)	< 0.001
Analgesic drugs use	16 (64.0%)	15 (15.8%)	< 0.001

### Postoperative complications within 1 week and inpatient days

Postoperative complications within 1 week and inpatient days are summarized in Table 4. There was no significant difference in the incidence of nausea and vomiting within postoperative day 7 between the two groups (*p* = 0.063). The incidence of postoperative fever in the poor sleep group (*p* = 0.016) was significantly higher than that in the Good sleep group. In addition, the length of hospital stay for patients in the poor sleep group was significantly prolonged (*p* < 0.001).

**Table 4:** Postoperative complications within 1 week and inpatient days (*n* = 120).

Items	Poor sleep quality group (*n* = 25)	Good sleep quality group (*n* = 95)	*p*
Nausea and vomiting	10 (40.0%)	19 (20.0%)	0.063
Fever (≥38.0 °C)	8 (32.0%)	12 (12.6%)	0.033
Hospital days	9.62 ± 3.61	7.35 ± 1.7	<0.001

### Correlation analysis

Univariate analysis revealed that preoperative sleep disturbance (PSQI as a continuous variable) was significantly associated with postoperative pain (OR = 1.379, 95% CI: 1.180–1.613, *p* < 0.001). Other variables, such as sex, age, BMI, marital status, smoking, alcohol consumption, comorbidities, surgical side, and responsible artery, showed no significant associations (*p* > 0.05) (Table 5).

**Table 5:** Univariate analysis of moderate-to-severe pain at postoperative day 7 (*n* = 120).

Influential factors	OR	95%CI_lower	95%CI_upper	*p*
Preoperative sleep disturbance	1.379	1.18	1.613	0
Gender	0.48	0.15	1.538	0.217
Age	1.011	0.969	1.055	0.611
BMI	0.951	0.825	1.096	0.487
Marital status	0.216	0.013	3.602	0.286
Smoking	0.553	0.117	2.618	0.455
Drinking	0.419	0.051	3.456	0.419
Comorbidity	2.094	0.818	5.365	0.123
Side (left)	1.444	0.566	3.689	0.442
Responsible artery				
AICA	0.495	0.189	1.297	0.152
PICA	2.392	0.841	6.8	0.102
VA & BA	0.989	0.198	4.934	0.99

AICA, anterior inferior cerebellar artery; PICA, posterior inferior cerebellar artery; VA, vertebral artery; BA, basilar artery.

Multivariate Logistic Regression Analysis, multivariate analysis adjusted for variables including age, sex, BMI, smoking, comorbidities, and surgical details (PICA). The results demonstrated that preoperative sleep quality (PSQI as a continuous variable) remained an independent risk factor for moderate-to-severe postoperative pain (OR = 1.368, 95% CI: 1.154–1.621, *p* < 0.001). None of the other variables showed statistical significance (Table 6).

**Table 6:** Logistic regression analysis of moderate-to-severe pain at postoperative day 7 (*n* = 120).

Influential factors	*β*	OR	95%CI_lower	95%CI_upper	*p*
Preoperative sleep disturbance	0.313	1.368	1.154	1.621	0
Gender	−0.628	0.534	0.102	2.795	0.457
Age	−0.005	0.995	0.943	1.049	0.842
BMI	−0.034	0.966	0.822	1.136	0.678
Smoking	−0.455	0.634	0.065	6.181	0.695
Comorbidity	0.65	1.916	0.596	6.152	0.275
PICA	0.476	1.609	0.431	6.003	0.479

**Figure 1:**
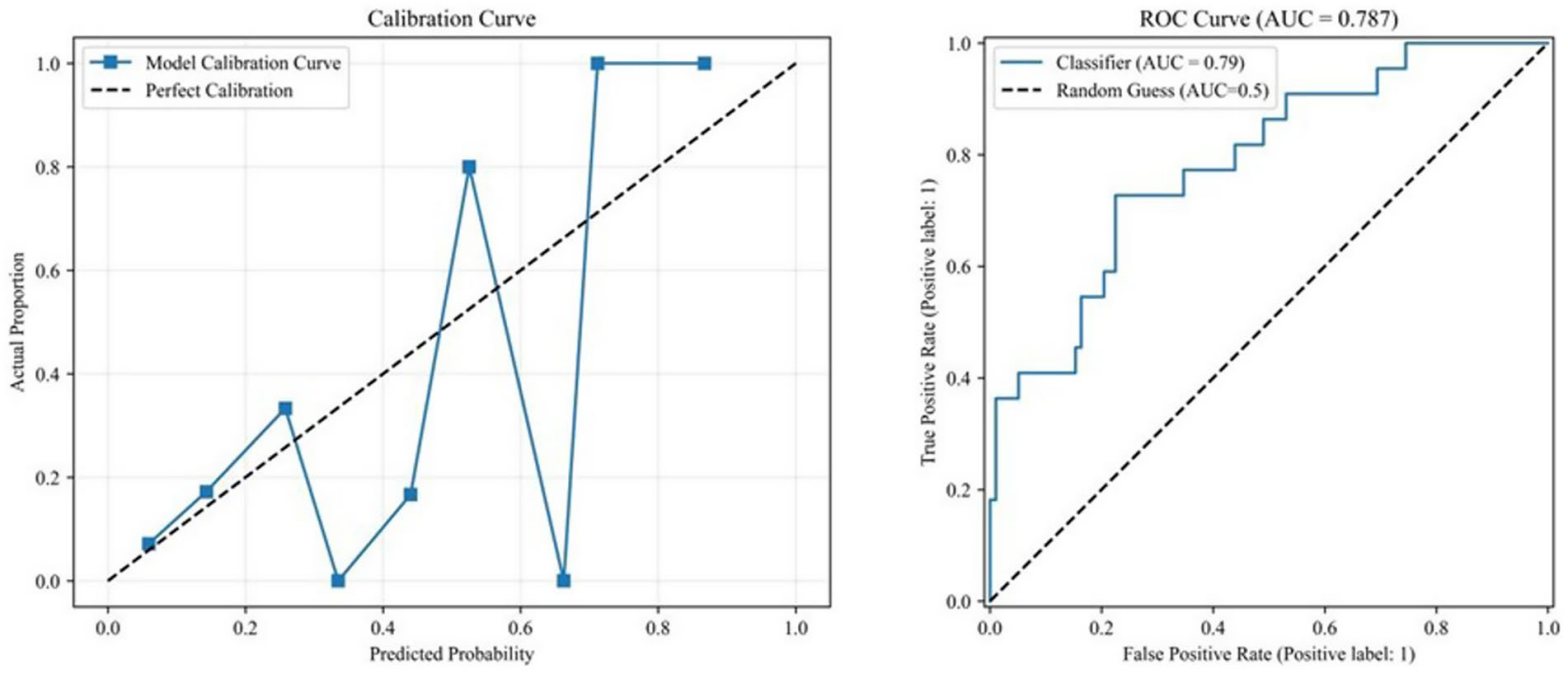
Calibration_and_ROC_Curves.

Model Performance, the primary analysis model (PSQI as a continuous variable) achieved an AUC of 0.787, indicating good discriminatory ability. The calibration curve showed that the predicted probabilities were generally consistent with the actual observed rates (Figure 1).

Sensitivity Analysis, after converting PSQI into a binary variable, the results remained robust:

PSQI ≥ 5: OR = 6.263, 95% CI: 2.127–18.442, *p* = 0.001, AUC = 0.774.

PSQI ≥ 7: OR = 6.419, 95% CI: 2.146–19.202, *p* = 0.001, AUC = 0.736.

This indicates that regardless of the coding method, preoperative sleep disturbance significantly increases the risk of postoperative pain (Table 7).

**Table 7:** Sensitivity analysis of PSQI coding methods on outcome association.

Items	PSQI coding protocol	critical value	OR	95%CI	*p*	AUC
Preoperative sleep disturbance	Continuous variable		1.368	1.154–1.621	0	0.787
Preoperative sleep disturbance	Binary variable	≥5	6.263	2.127–18.442	0.001	0.774
Preoperative sleep disturbance	Binary variable	≥7	6.419	2.146–19.202	0.001	0.736

## Discussion

This study investigated the correlation between preoperative sleep disturbances and postoperative pain in patients with hemifacial spasm (HFS), providing critical evidence for clinical intervention. The results demonstrated a significant association between poor preoperative sleep quality (higher PSQI scores) and the severity of postoperative pain. Multivariate analysis showed that after adjusting for potential confounding factors including age, gender, BMI, smoking history, comorbidities, and surgical details, each 1-point increase in the preoperative PSQI score was associated with a 36.8% increased risk of moderate-to-severe pain 1 week after surgery (adjusted OR = 1.368, 95% CI: 1.154–1.621, *p* < 0.001).

Sensitivity analysis further confirmed the robustness of this association. When using PSQI cutoff values of ≥5 and ≥7, the risk of moderate-to-severe pain in patients with poor sleep quality was 6.263 times (95% CI: 2.127–18.442) and 6.419 times (95% CI: 2.146–19.202) that of the control group, respectively, with both results being statistically significant (*p* = 0.001). These findings are consistent with previous reports in other surgical fields, further supporting the significant impact of preoperative sleep status on postoperative pain.

These results align with previous findings in other surgical disciplines, further confirming the significant impact of preoperative sleep status on postoperative pain (8). Sleep disturbances may activate neuroinflammatory responses and disrupt hypothalamic–pituitary–adrenal (HPA) axis function, thereby heightening pain sensitivity. For HFS patients, chronic facial muscle spasms not only impair sleep but also induce psychological stress, creating a vicious cycle between sleep disturbances and pain perception (5). Preoperative sleep deprivation or poor sleep quality may compromise neuroregulation and immune function during postoperative trauma response, exacerbating pain through homeostatic imbalance.

From a clinical perspective, these findings underscore the importance of incorporating sleep assessment into the perioperative management of HFS patients—a population already vulnerable to neural dysfunction. First, perioperative management should prioritize sleep assessment in HFS patients. Those identified with sleep disturbances (PSQI ≥7) warrant timely evaluation and multimodal interventions, including cognitive behavioral therapy for insomnia and judicious pharmacological support when indicated (21). Improving preoperative sleep quality may reduce postoperative pain intensity, decrease analgesic requirements, and enhance recovery outcomes and patient satisfaction.

Second, this study highlights several research directions. While establishing the association between preoperative sleep disturbances and postoperative pain, the underlying biological mechanisms require further exploration. Future investigations should examine molecular pathways linking sleep disturbances to pain perception and differential impacts of specific sleep stage disruptions (22–24).

Notably, secondary observations revealed that the poor sleep group experienced higher rates of postoperative fever and prolonged hospitalization. This phenomenon may be related to sleep-related immune dysfunction predisposing patients to inflammation, suggesting that clinical attention should extend beyond pain management to encompass other sleep-associated postoperative complications. One study showed that patients with preoperative sleep disturbances had significantly longer length of stay than those without sleep disturbances (25).

Nevertheless, our study is limited by its single-disease, single-procedure design, which may restrict the generalizability of the findings. Additionally, the reliance on self-reported measures for sleep quality (PSQI) and pain intensity (NRS) is subject to potential recall and reporting biases. Information regarding prior treatment for sleep disorders was not collected, which might represent a confounding factor. Future multicenter studies involving more diverse surgical populations while controlling for procedure-specific confounders (e.g., CSF leakage, pneumocephalus) are needed to validate these results and further elucidate the underlying mechanisms.

In conclusion, this study establishes a significant association between preoperative sleep disturbances and postoperative pain in HFS patients, providing a theoretical foundation for developing targeted perioperative interventions.

## Data Availability

The raw data supporting the conclusions of this article will be made available by the authors, without undue reservation.
